# *Citrullus mucosospermus* Extract Exerts Protective Effects against Methionine- and Choline-Deficient Diet-Induced Nonalcoholic Steatohepatitis in Mice

**DOI:** 10.3390/foods13132101

**Published:** 2024-07-01

**Authors:** Sun Young Park, Ji Eun Kim, He Mi Kang, Ki Ho Park, Byoung Il Je, Ki Won Lee, Dae Youn Hwang, Young Whan Choi

**Affiliations:** 1 Institute of Nano-Bio Convergence, Pusan National University, Busan 46241, Republic of Korea; sundeng99@pusan.ac.kr; 2 Department of Biomaterials Science (BK21 FOUR Program)/Life and Industry Convergence Research Institute/Laboratory Animals Resources Center, Pusan National University, Miryang 50463, Republic of Korea; prettyjiunx@naver.com (J.E.K.); pujihao@naver.com (K.H.P.); dyhwang@pusan.ac.kr (D.Y.H.); 3 Department of Horticultural Bioscience/Life and Industry Convergence Research Institute, College of Natural Resources and Life Science, Pusan National University, Miryang 50463, Republic of Korea; mimi2965@naver.com (H.M.K.); bije@pusan.ac.kr (B.I.J.); 4 Natural Products Convergence R&D Division, Kwangdong Pharm. Co., Ltd., Seoul 08381, Republic of Korea; wish2k@nate.com

**Keywords:** *Citrullus mucosospermus*, nonalcoholic steatohepatitis, methionine and choline deficiency

## Abstract

In recent years, there has been increasing interest in exploring the potential therapeutic advantages of *Citrullus mucosospermus* extracts (CME) for nonalcoholic steatohepatitis (NASH). In this study, we investigated the therapeutic effects of CME on NASH using a mice model. High-performance liquid chromatography (HPLC) was employed to identify cucurbitacin E and cucurbitacin E-2-O-glucoside from the CME. Although CME did not significantly alter the serum lipid levels in methionine- and choline-deficient (MCD) mice, it demonstrated a protective effect against MCD diet-induced liver damage. CME reduced histological markers, reduced alanine transaminase (ALT) and aspartame transaminase (AST) levels, and modulated key NASH-related genes, including *C/EBPα*, *PPARγ*, *Fas*, and *aP2*. In addition, CME was found to restore hormone-sensitive lipase (*HSL*) and adipose triglyceride lipase (*ATGL*) activity, both crucial for fat catabolism, and reduced the levels of pro-inflammatory cytokines. Furthermore, CME demonstrated the potential to mitigate oxidative stress by maintaining or enhancing the activation and expression of nuclear factor erythroid 2-related factor 2 (*Nrf2*) and superoxide dismutase (*SOD*), both pivotal players in antioxidant defense mechanisms. These findings underscore the promising therapeutic potential of CME in ameliorating liver damage, inflammation, and oxidative stress associated with NASH.

## 1. Introduction

Excess fat accumulates in the liver, primarily in the form of triglycerides, which can lead to two main types of fatty liver diseases. The first type is alcoholic fatty liver disease, resulting from excessive alcohol consumption, and the second is nonalcoholic fatty liver disease (NAFLD), which is attributed to factors such as obesity, diabetes, hyperlipidemia, and medication use [1,2]. Alcoholic fatty liver disease is caused by the overconsumption of alcohol, leading to the accumulation of fatty acids (generated during the breakdown of alcohol) in the liver cells. This condition predominantly resolves naturally, without progression to liver cell damage or fibrosis. NAFLD, a growing concern affecting individuals with minimal alcohol consumption, is often associated with obesity, diabetes, high cholesterol levels, hypertension, autoimmune issues, and certain medications [3,4]. In addition, NAFLD is mediated by a complex interplay of several factors, including adipose tissue hormones, insulin resistance, dietary choices, the gut microbiome, genetic predispositions, and lifestyle habits. Notably, if left unchecked, NAFLD can progress to fatty liver inflammatory diseases such as nonalcoholic steatohepatitis (NASH), with liver cell damage or fibrosis. Unhealthy indulgence and excessive calorie intake contribute to liver fat accumulation. This overflow burdens both the peripheral and liver tissues, triggering inflammatory pathways. The liver acts as a metabolic master, orchestrating interactions between peripheral fat and the gut through cytokine messengers [5,6,7,8]. Free fatty acid flooding from peripheral fat overwhelms the liver cells, disrupting mitochondrial and peroxisomal functions and causing oxidative stress. Elevated reactive oxygen species (ROS) wreak havoc on liver cells, triggering macrophage infiltration. The resulting chronic inflammation can lead to a metabolic decline and fibrosis [9,10]. Currently, there is no specific treatment for NAFLD, and clinical management primarily involves lifestyle modifications, such as weight control, dietary adjustments, and increased physical activity. However, when more aggressive interventions are required, drug therapy is considered. Therefore, the development of safe and effective drugs to delay NAFLD progression is a central focus of current research in this field [11,12,13]. NAFLD is a growing public health concern characterized by the accumulation of fat in the liver without significant alcohol consumption. It includes a spectrum of liver conditions, ranging from simple steatosis to NASH, which can progress to cirrhosis and liver cancer. The increasing prevalence of NAFLD is closely linked to the global rise in obesity, diabetes, hyperlipidemia, and metabolic syndrome [14]. Despite the increasing prevalence of NAFLD, there are currently no approved pharmacological treatments specifically targeting this condition. However, extensive research efforts are ongoing to identify potential therapeutic targets and develop novel interventions. Promising areas of investigation include regulating lipid metabolism, addressing insulin resistance, targeting inflammation, modulating oxidative stress, and influencing the gut microbiome. Drugs that modulate lipogenesis, lipid oxidation, and lipid transport pathways could alleviate hepatic steatosis, a hallmark of NAFLD [15,16,17]. Improving insulin sensitivity through agents such as metformin and thiazolidinediones is being explored for their therapeutic potential. Chronic inflammation, a driving force behind the progression of NAFLD to more severe forms like NASH, is being targeted through anti-inflammatory agents [18]. Additionally, antioxidants and agents that enhance the body’s antioxidant defenses, particularly those targeting the *Nrf2* pathway, are potential therapeutic strategies. Emerging evidence suggests a link between gut dysbiosis and NAFLD pathogenesis, prompting investigations into prebiotics, probiotics, and fecal microbiota transplantation as potential interventions to restore gut homeostasis. While lifestyle modifications remain the mainstay of NAFLD management, the development of effective pharmacological therapies is a critical area of research, with numerous ongoing clinical trials investigating various therapeutic targets and approaches [18,19,20]. *Citrullus lanatus* (Sweet Dessert Watermelon) is a gourd plant that originates in tropical and subtropical countries and is among the most widely consumed vegetable crops worldwide [21,22]. Watermelons contain various nutrients such as water, carotenoids, organic acids, vitamins, carbohydrates, fats, crude fiber, amino acids, nucleotides, citrulline, arginine, lycopene, and cucurbitacins. These nutrients contribute to the therapeutic and pharmacological effects of watermelons. *C. lanatus* belongs to the Cucurbitaceae family and the genus *Citrullus*, which comprises seven species including *C. lanatus* (Thunb.) Matsum. & Nakai, *C. mucosospermus* (Fursa), *C. amarus* (Schrad.), *C. colocynthis* (L.) Schrad., *C. naudinianus* (Sond.) Hook, *C. rehmii* De Winter, and *C. ecirrhosus* Cogn. Among these, *C. mucosospermus* (Egusi Watermelon) is native to Africa and is cultivated primarily for seed consumption [23,24,25,26,27]. Seeds of this watermelon species lack a hard coat, which allows them to be eaten raw. However, its white fruit pulp is too bitter for consumption. Recent studies on *C. mucosospermus* have focused on its antioxidant and anti-inflammatory effects, anticancer properties, antimicrobial activities, blood sugar regulation, cholesterol control, and potential for weight loss. This species, known as egusi, is a staple in African traditional cuisine and shows promise for various applications as research continues to uncover its potential benefits [27,28,29,30]. Both *C. lanatus* and *C. mucosospermus* belong to the *Citrullus* genus, but differ in seed edibility, taste of the fruit pulp, nutritional content, and utilization in culinary practices. *C. lanatus* is popular worldwide for its sweet and tender fruits, which are consumed raw or used in various dishes such as juices, salads, and desserts. On the other hand, *C. mucosospermus* is characterized by a bitter fruit pulp, and its seeds are the primary focus for consumption in traditional African cuisine [31,32,33,34]. Ongoing studies on *C. mucosospermus* have revealed its potential efficacy in various fields, making it an emerging subject of interest. Ciprofloxacin, the positive control used in this study, has been established to effectively treat NASH. Its action relies on a receptor known as PPARα, which plays a crucial role in various biological processes, including fat metabolism, inflammation, and fibrosis [35,36,37,38,39]. An MCD diet serves as a dietary model for studying NASH, inducing the disease through alterations in lipid metabolism in both adipose tissue and the liver. A deficiency in methionine and choline, essential nutrients for lipid metabolism, and an MCD diet trigger key pathological features of NASH, including steatosis, inflammation, liver damage, and fibrosis. This dietary model is valuable for investigating NASH’s pathogenesis and identifying potential therapeutic targets.

In this study, C57BL/6 mice were exposed to an MCD regimen supplemented with daily oral gavage of CME (extract) at varying doses over 4 weeks. The main objective of this study was to explore the mechanisms underlying the protective effects of CME against MCD-induced NASH using a C57BL/6 mouse model. This study aimed to elucidate the therapeutic potential of CME and the underlying pathways in MCD-induced NASH. By unraveling these mechanisms, researchers can identify novel therapeutic targets and advance the development of novel treatments for NAFLD/NASH and NASH.

## 2. Materials and Methods

### 2.1. CM Cultivation and Extract

In this study, *Citrullus mucosospermus* (CM) extract prepared from plants cultivated at the Pusan National University Farm was utilized. Planting commenced in mid-April, and ripe fruits were harvested at maturity. The ripe fruits were cut into pieces of approximately 3 cm in width and length without being separated into flesh, rind, and seeds. These pieces were then lyophilized using a freeze dryer (Ilshin, Dongducheon, Republic of Korea) and pulverized into a fine powder using a blender (Hanil mixer, Bucheon, Republic of Korea). The resulting powder was then sieved through a 30-mesh sieve, and the resulting extract was used in the study. Before the experiment, 10 g of powdered CM was extracted with 60 mL of distilled water using a sonicator (JAC Ultrasonic, Seoul, Republic of Korea) for 1 h at room temperature at a water-to-powder ratio of 1:6. The extract was subsequently filtered through Whatman No. 2 filter paper and concentrated using a rotary evaporator (Heidolph, Seoul, Republic of Korea) to yield 4.46× *g* of water extract. This *Citrullus mucosospermus* extract (CME) was stored in a glass bottle at −20 °C until required.

### 2.2. HPLC Analysis

In this study, a high-performance liquid chromatography (HPLC) system (Agilent 1100, Santa Clara, CA, USA) equipped with an autoinjector, and column temperature controller was used to quantify cucurbitacin E and cucurbitacin E-2-O-glucoside levels the CME. Separation was achieved using a reversed-phase Luna C18 column (150 mm × 4.6 mm I.d., 5 μm particle diameter, Pheomenex, Torrance, CA, USA). The mobile phase consisted of 0.01% formic acid (A) and acetonitrile (B) in water. The gradient elution program commenced at 40% B, was maintained for 30 min, and then increased to 100% B over 10 min. For CME analysis, a 20 μL injection volume was used with a flow rate of 0.5 mL/min and a constant column temperature of 30 °C.

### 2.3. Non-Alcoholic Steatohepatitis Animal Study

**Figure 1:**
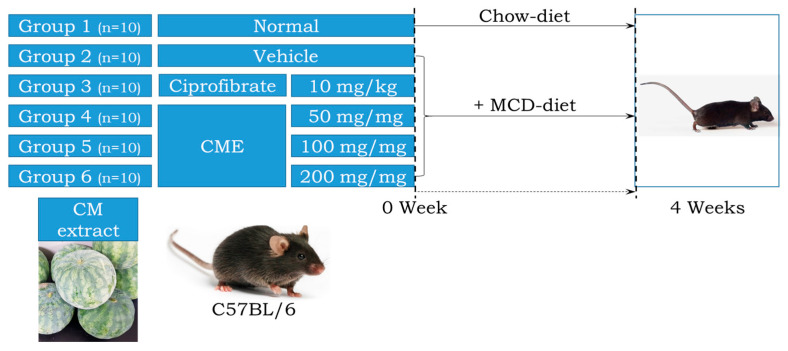
Experimental design for investigating the effects of *Citrullus Mucosospermus* extract on MCD Diet.

All animal procedures adhered to the ethical principles outlined in the Pusan National University Institutional Animal Care and Use Committee (PNU-IACUC) guidelines (Approval Number PNU-2022-0139). Housing and husbandry were maintained at the Pusan National University Laboratory Animal Resources Center, a facility accredited by the Korea Food and Drug Administration (KFDA) and the Association for Assessment and Accreditation of Laboratory Animal Care (AAALAC) International. Seven-week-old male C57BL/6 mice (n = 60) from Samtako Bio-Korea Inc. (Osan, Republic of Korea) were randomly assigned to either the control group or MCD diet group (10 animals each). The control group received a standard irradiated chow diet, while the MCD group received an MCD diet (Saeronbio, Uiwangsi, Republic of Korea). Within the MCD group, further sub-groups were established: MCD + Po received 10 mg/kg body weight Ciprofibrate (ciprofloxacin) daily via oral administration. MCD + CME 50, 100 + 200 received 50 mg/kg, 100 mg/kg, and 200 mg/kg body weight CME daily via oral administration, respectively. The MCD + Ve mice received the same volume of distilled water daily via oral administration (Figure 1). Throughout the four-week study period, the mice were housed under controlled conditions in a specific pathogen-free (SPF) environment. They were maintained on a 12 h light/dark cycle, at a constant temperature of 23 ± 2 °C, and with appropriate humidity levels of 50 ± 10%. Body weight was measured twice weekly and food intake and water consumption were monitored weekly according to the KFDA guidelines. At the end of the experiment, the mice were euthanized using CO_2_ gas and their tissue samples were collected and stored at −70 °C for further analysis.

### 2.4. Serum Biochemical Analysis for NASH

Following the final administration of the respective treatments, all C57BL/6 mice were subjected to an 8 h fast for metabolic stabilization. Subsequently, the animals were euthanized using a humidified CO_2_ gas protocol. Whole blood was carefully extracted from the abdominal veins using sterile syringes and needles. To obtain serum for biochemical analyses, whole blood samples were centrifuged at 1500× *g* for 15 min. The resulting serum was analyzed for various biochemical components, including ALT, AST, total cholesterol (TC), triglyceride (TAG), high-density lipoprotein (HDL), and low-density lipoprotein (LDL). The analysis was performed using a reputable automated serum analyzer (Hitachi 747, Tokyo, Japan, or equivalent) following the manufacturer’s protocols. All assays were performed in duplicate using fresh serum samples to ensure accurate and reliable results.

### 2.5. Histopathological Analysis for NASH

The method for assessing lipid accumulation in liver tissue involved harvesting fresh liver tissues immediately after sacrificing the experimental animals and rapidly frozen in isopentane cooled with liquid nitrogen. Frozen tissues were then sectioned into 8–10 μm thickness using a cryostat. Frozen sections were stained with Oil Red O staining solution for approximately 10 min, followed by rinsing for 2 min each in 85% propylene glycol solution and distilled water. Following rinsing, the sections underwent counterstaining with hematoxylin for 1 min to stain the nuclei. Subsequently they were dehydrated and mounted to prepare the final slides. Additionally, for routine histological examination, distinct liver tissue samples were fixed in 10% neutral buffered formalin for 48 h. These samples then underwent dehydration, embedding in paraffin, and sectioning into 4 μm thickness. The sections were stained with hematoxylin and eosin (H&E) staining. All stained slides were observed at 200× and 400× magnification using a Leica Application Suite microscope (Leica Microsystems, Herbrugg, Switzerland), and their images were captured.

### 2.6. Quantitative Real Time–Polymerase Chain Reaction (qRT-PCR) Analysis for NASH

Total RNA was extracted from each liver tissue sample using the RNeasy Mini Kit (Qiagen, Hilden, Germany) following the manufacturer’s protocol. Briefly, homogenized tissue lysates were prepared using a Polytron homogenizer (Kinematica AG, Luzern, Switzerland) and centrifuged at 10,000× *g* for 15 min to isolate RNA. RNA concentration was quantified using a NanoDrop spectrophotometer (Thermo Fisher Scientific, Waltham, MA, USA). To analyze gene expression, cDNA was synthesized from 4 μg of mRNA using the SuperScript II Reverse Transcriptase kit (Invitrogen, Carlsbad, CA, USA) according to the manufacturer’s instructions. Subsequently, quantitative real-time PCR (qPCR) was performed on the resulting cDNA using SYBR Green PCR Master Mix (Applied Biosystems, Foster City, CA, USA) and specific primers for genes related to acrolein metabolism (listed in Supplementary Table S1).

### 2.7. Western Blotting Analysis for NASH

Total protein was extracted from the liver tissue of each mouse using a commercially available protein extraction solution, following the manufacturer’s instructions. Following centrifugation to separate cellular debris, protein concentrations were determined using a BCA protein assay kit (SMART™ Bicinchoninic Acid Protein Assay Kit). Equal amounts of protein were separated by sodium dodecyl sulfate-polyacrylamide gel electrophoresis on a 4–20% gradient gel for 2 h and subsequently transferred onto nitrocellulose membranes. The membranes were blocked and incubated overnight at 4 °C with primary antibodies targeting specific proteins of interest, including lipid metabolism, hormone-sensitive lipase (*HSL*, Cell Signaling #4107, 1:1000, Cell Signaling Technology, Beverly, CA, USA), phosphorylated HSL (*p-HSL*, Cell Signaling #45804, 1:1000, Cell Signaling Technology), and adipose triglyceride lipase (*ATGL*, #2138, 1:1000, Cell Signaling Technology). Additionally, oxidative stress markers such as superoxide dismutase (*SOD*, ab13498, 1:1000, Abcam, Cambridge, UK) and nuclear factor E2-related factor 2 (*Nrf2*, ab137550, 1:1000, Abcam), along with Nrf2 activation factors such as phosphorylated *Nrf2* (*p-Nrf2*, PA5-67520, 1:1000, Invitrogen Co., Carlsvad, CA, USA), were assessed. Glyceraldehyde 3-phosphate dehydrogenase (*GAPDH*) served as the loading control. Following washes, membranes were incubated with horseradish peroxidase (*HRP*)-conjugated secondary antibody for 1 h at room temperature. Chemiluminescent detection reagent (Amersham ECL Select) was used to visualize protein bands, and chemiluminescence signals were captured using an imaging system (FluorChemi^®^FC2). Protein band intensities were quantified by densitometry analysis using image analysis software (ImageJ software v 1.47). The intensity of each protein band was calculated as a ratio relative to the intensity of the *GAPDH* loading control.

### 2.8. Statistical Analysis

The statistical significance of the differences between groups was assessed by one-way analysis of variance (ANOVA) performed using a statistical software package (SPSS for Windows, Release 10.10, or equivalent). For significant ANOVA results, post hoc comparisons were conducted using Tukey’s test to identify specific group differences. Data are presented as mean ± standard deviation (SD). A *p*-value less than 0.05 (*p* < 0.05) was considered statistically significant.

## 3. Results

### 3.1. Quantification of Cucurbitacins in the CME 

**Figure 2:**
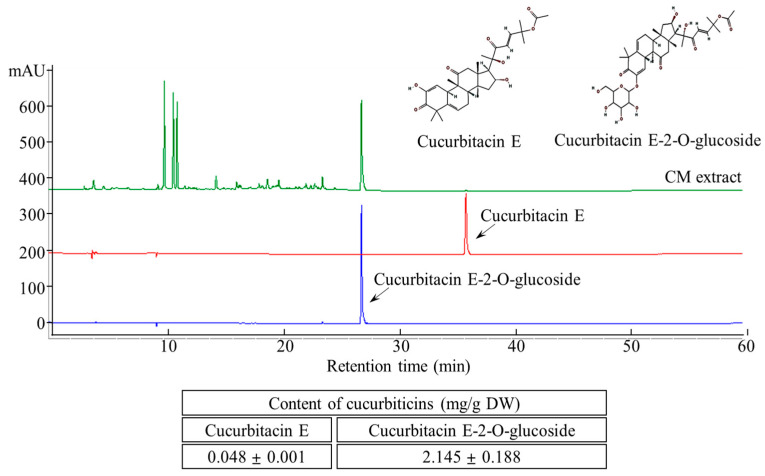
HPLC chromatogram of cucurbitacin E and cucurbitacin E-2-O-Glucoside content in CM extracts.

The two major cucurbitacins, cucurbitacin E and cucurbitacin E-2-O-glucoside, were well separated using HPLC (Figure 2). Simultaneous quantification of these compounds was performed by UV detection at 254 nm. The cucurbitacin E and cucurbitacin E-2-O-glucoside contents of the aqueous CME were 0.048 ± 0.001 and 2.145 ± 0.188 mg/g, respectively.

### 3.2. Effect of CME on Body Weight, Liver Weight, and Relative Liver Weight of Mice

**Figure 3:**
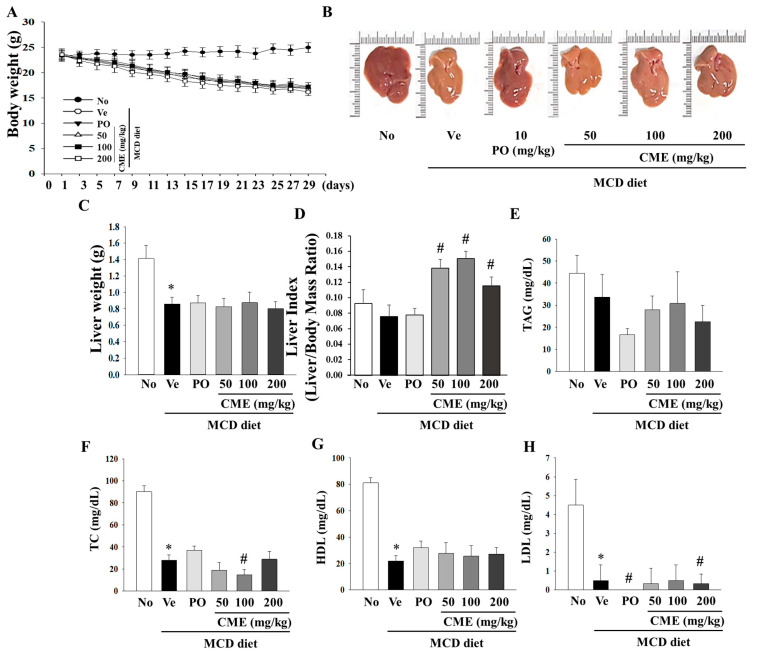
Effects of CME on body weight, liver weight, and lipid deposition in MCD diet-fed C57BL/6 mice. Examining the impact of CME on body weight, liver weight, and relative liver weight (%) in mice subjected to chow or methionine- and choline-deficient (MCD) diets over a 4-week period. CME was administered at doses of 50 mg, 100 mg, and 200 mg/kg/day, alongside ciprofibrate at 10 mg/kg/day. (**A**) Effect of CME on body weight. (**B**) Effect of CME on liver morphology. (**C**) Effect of CME on liver weight. (**D**) Effect of CME on liver index. Analysis of serum TAG level (**E**) and TC (**F**), HDL, (**G**) and LDL (**H**) level. Results are presented as the mean ± SE. * *p* < 0.05 indicates significance compared to the No control, and # *p* < 0.05 indicates significance compared to the vehicle group in C57BL/6 mouse liver tissues.

This study was designed to investigate the potential therapeutic effects of CME on NASH using an animal model. The experimental setup involved a control group subjected to a standard diet, while the NASH model group was fed a diet deficient in methionine and choline to induce NASH. CME supplementation spanned over 4 weeks with varying doses of 50, 100, and 200 mg/kg body weight. As expected, the MCD diet-induced a notable reduction in body weight, liver weight, and liver weight-to-body weight ratio in NASH mice, which persisted even after 4 weeks (Figure 3A–C). Interestingly, CME-treated mice exhibited distinct statistical differences in these effects (Figure 3A). The livers of the control group mice displayed a characteristic smoothness with sharp edges and a dark reddish color. In contrast, the livers of the MCD group mice appeared smaller, exhibited a lighter yellow hue, and displayed a greasy surface with blunt edges compared with those of the control group. Remarkably, the livers of mice in the CME and positive control (MCD-Po) groups exhibited minimal differences in volume compared to those in the MCD group. However, their color gradually transitioned towards a darker red color with smooth surfaces and sharp edges (Figure 3B,C). Additionally, the positive control, ciprofloxacin, yielded distinct outcomes in both body weight and liver indices. Notably, CME supplementation significantly increased the liver index (liver weight/body weight ratio) in mice fed the MCD diet (Figure 3D). Further analysis of the biochemical aspects revealed that the MCD diet-fed mice exhibited lower serum total cholesterol levels than the control group. The MCD diet also led to significant reductions in serum TAG, TC, HDL, and LDL levels. However, these changes were not significantly altered by CME supplementation in the low-, medium-, and high-dose groups (Figure 3E–H).

### 3.3. CME Modulates Lipid Metabolism, Inflammatory Response, and Oxidative Stress in the Liver

**Figure 4:**
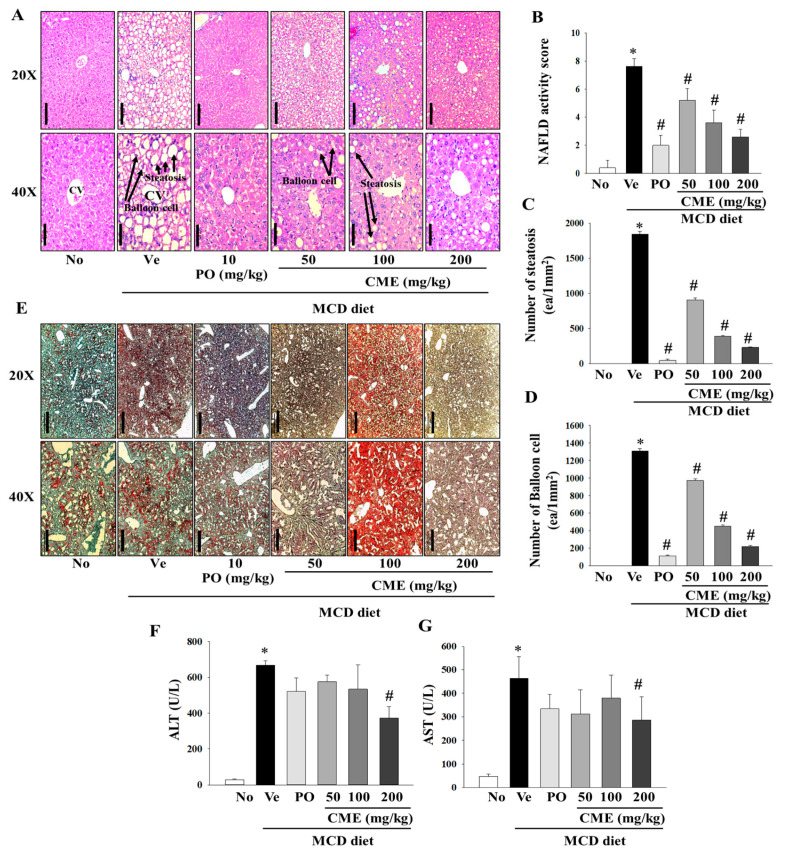
Effect of CME on hepatic steatosis and liver injury in MCD diet-fed C57BL/6 mice. (**A**) The H&E staining of the liver tissues (Scale bar; 100 mm (20×), 50 mm (40×)). (**B**) Statistical analysis of activity score of nonalcoholic fatty liver disease. (**C**) Statistical analysis of steatosis. (**D**) Statistical analysis of balloon cells. (**E**) Oil O Red staining of the liver tissues (Scale bar; 500 mm (20×), 200 mm (40×)). Analysis of serum ALT (**F**) and AST (**G**) levels. Results are presented as the mean ± SE. * *p* < 0.05 indicates significance compared to the No control, and # *p* < 0.05 indicates significance compared to the vehicle group in C57BL/6 mouse liver tissues.

In this comprehensive study, we aimed to investigate the intricate effects of CME extract on liver damage and hepatic fat accumulation in mice fed an MCD diet. Histopathological staining of liver tissues provide a nuanced understanding of the morphological alterations induced by an MCD diet and the potential mitigating influence of CME. Histological examination, particularly through H&E staining, vividly portrayed profound changes in liver morphology in MCD-fed mice, highlighting a notable increase in both the number and volume of lipid droplets. In contrast, mice treated with MCD-CME and MCD-Po exhibited a discernible reduction in the size and quantity of lipid droplets, as shown in Figure 4A. Furthermore, parameters such as the NAFLD activity score, number of steatotic cells, and number of balloon cells depicted a concentration-dependent decline in the MCD-CME-fed mice, showing a promising trend towards improvement compared to the MCD-fed mice (Figure 4B–D). Notably, these effects were consistently significant in MCD-Po-fed mice, underscoring the potential efficacy of MCD-CME in mitigating the histological markers of liver damage and fat accumulation. Complementary to H&E staining, Oil Red O staining further confirmed the altered liver morphology in MCD-fed mice, accentuating the increased presence of lipid droplets. A digital image analysis of Oil Red O not only substantiated these findings but also demonstrated enhanced sensitivity and specificity in detecting steatosis compared to the traditional visual evaluation of H&E staining. The supplementation with CME resulted in a pronounced reduction in both the number and size of lipid droplets in the liver cells, mirroring the effects observed in the positive control group (Figure 4E). Regarding biochemical parameters, serum ALT and AST levels, which are often indicative of liver function, were significantly elevated in MCD-fed mice compared to those in the control group. Interestingly, the CME-supplemented MCD-CME mice yielded a noteworthy attenuated these increases, suggesting a protective effect against MCD diet-induced liver damage. In particular, a significant decrease in serum AST levels was observed in the MCD-Po group, further emphasizing the potential of CME to mitigate hepatic injury (Figure 4F,G).

### 3.4. Molecular Mechanisms Underlying the Protective Effects of CME in NASH

**Figure 5:**
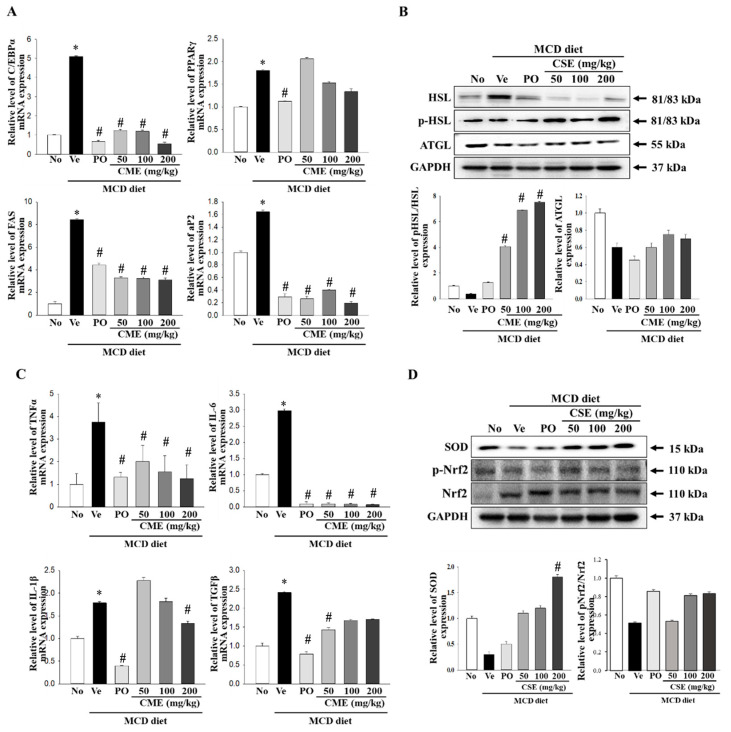
Effect of CME on hepatic fat metabolism and inflammation in MCD diet-fed C57BL/6 mice. (**A**) mRNA levels of *PPAR-γ*, *C/EBPα*, *Fas*, and *aP2* in liver tissues from control, MCD-fed mice, and MCD-CME-fed mice are shown. (**B**) Western blot analysis reveals protein expression of *p-HSL, HSL*, and *ATGL* in each group. Each blot represents at least three independent samples. (**C**) mRNA levels of pro-inflammatory cytokines *TNF-α*, *IL-6*, *IL-1β*, and *TGF-β* in liver tissues are presented. (**D**) Western blot analysis depicts protein expression of antioxidant enzyme SOD and activated *Nrf2* (p-Nrf2) relative to total Nrf2 in each group. Each blot represents at least three independent samples. Data are presented as mean ± SEM for 10 mice per group (* *p* < 0.05 vs. control group, # *p* < 0.05 vs. vehicle group in C57BL/6 mouse liver tissues).

In a NASH model, increased C/EBP expression suppresses intrahepatic inflammation and promotes fibrosis. Concurrently, alterations in visceral fat and adiponectin levels, mediated by *PPARγ*, are associated with the amelioration of steatohepatitis in NASH patients. Moreover, the overactivity of *Fas*, an enzyme involved in fatty acid synthesis, can impact abnormal fat accumulation and inflammation in patients with NASH when it is overly active. In NASH, abnormal hepatic fat accumulation is linked to impaired AP2 function [16,17,40]. To further explore the mechanism of action, we examined the expression of these factors using real-time PCR. The results, presented in Figure 5A, revealed a significant increase in the relative mRNA levels of *C/EBPα*, *PPARγ*, *Fas*, and *aP2* in the MCD-fed group compared to the control group. Conversely, MCD-CME-fed mice displayed decreased mRNA levels compared with MCD-fed mice (Figure 5A). Hormone-sensitive lipase (*Hsl*) and adipose triglyceride lipase (*Atgl*) play crucial roles in regulating fat breakdown in the adipose tissue by breaking down neutral fats within fat cells into glycerol and fatty acids. An examination of the expression of the enzymes involved in lipid metabolism revealed a decrease in their levels in MCD-fed mice compared to the control group. Remarkably, CME supplementation restored these activities, leading to an increase in both *Hsl* phosphorylation and *Atgl* protein expression (Figure 5B). *TNF-α*, *IL-6*, *IL-1β*, and TGF-β are key cytokines that regulate inflammatory responses. In the context of NASH, they exacerbate inflammatory reactions, leading to hepatocellular damage, fibrosis, and the progression to liver cirrhosis [41,42,43]. Real-time PCR was utilized in this study to assess their expression, with Figure 5C illustrating a notable rise in the relative mRNA levels of *TNF-α*, *IL-6*, *IL-1β*, *and TGF-β* in the MCD diet group compared to the control group. Conversely, mice receiving MCD-CME exhibited an overall reduction in mRNA levels compared to the MCD-fed group (Figure 5C). NASH disrupts the delicate balance between oxidative stress and antioxidant defense mechanisms, with *Nrf2* and *SOD* playing crucial roles in this interaction. *Nrf2*, a master regulator of antioxidant responses, activates the expression of antioxidant enzymes such as *SOD* to combat free radical damage. In NASH, excessive fat accumulation and oxidative stress impair *Nrf2* activity and *SOD* expression. Our study revealed that mice with MCD diet-induced NASH exhibited decreased Nrf2 phosphorylation and *SOD* expression (Figure 5D). Notably, cotreatment with MCD-CME maintained or even enhanced both *Nrf2* activation and *SOD* expression, suggesting a potential therapeutic benefit in mitigating oxidative stress in patients with NASH.

## 4. Discussion

Previous phytochemical studies on *C. mucosospermus* have identified various cucurbitacin derivatives, including cucurbitacin E and its glycosidic forms such as cucurbitacin E-2-O-glucoside. These compounds, belonging to the highly oxidized tetracyclic triterpenoids known as cucurbitacins, are widely distributed in the *Cucurbitaceae* family. Cucurbitacins exhibit diverse pharmacological properties, including hepatoprotective, anti-inflammatory, anti-cancer, and anti-diabetic effects [38,39,44]. Cucurbitacin E, in particular, has demonstrated potent antioxidant capacities and anti-inflammatory properties by inhibiting the production of pro-inflammatory cytokines like TNF-α and IL-6. Although less explored than its aglycone form, cucurbitacin E-2-O-glucoside, the primary component in our CME, has promising therapeutic potential. Further exploration of cucurbitacin glycosides from *Citrullus* species may reveal novel bioactive compounds and elucidate their structure–activity relationships. In addition to cucurbitacins, *C. mucosospermus* contains phenolic acids, flavonoids, and other terpenoids [45,46]. These secondary metabolites have been reported to inhibit adipogenesis, promote weight loss, and improve lipid profiles in obesity-related models. For instance, phenolic compounds such as chlorogenic acid and flavonoid glycosides inhibit preadipocyte differentiation, whereas terpenoids such as lycopene and cucurbitane triterpenoids activate fatty acid oxidation and suppress lipogenesis [47,48]. The synergistic effects of these diverse secondary metabolites in *C. mucosospermus* may contribute to its antioxidant, anti-inflammatory, and lipid metabolism-regulatory effects, potentially offering therapeutic benefits in NAFLD.

This study demonstrated that CME supplementation significantly ameliorated hepatic steatosis and inflammation in MCD diet-fed mice. Specifically, CME treatment resulted in a dose-dependent reduction in hepatic lipid accumulation, as evidenced by histopathological analyses and lipid quantification assays. Mice treated with CME exhibited reduced hepatic steatosis, ballooning, and lobular inflammation scores, which are key histological features of NAFLD/NASH. These findings suggest that CME exerts hepatoprotective effects by mitigating hepatic lipid accumulation and inflammation. At the molecular level, CME treatment modulated the expression of key genes involved in lipid metabolism, inflammation, and oxidative stress. The mRNA levels of lipogenic genes such as PPAR-γ, C/EBPα, and Fas were significantly downregulated in the livers of CME-treated mice compared to MCD-fed controls. Conversely, the expression of lipolytic genes such as HSL and ATGL was upregulated, indicating enhanced lipid catabolism. Additionally, CME treatment led to a significant reduction in the mRNA levels of pro-inflammatory cytokines TNF-α, IL-6, IL-1β, and TGF-β, which are known to play pivotal roles in the pathogenesis of NASH. Western blot analyses further corroborated these findings, revealing increased protein expression of the antioxidant enzyme SOD and activated Nrf2 (p-Nrf2) in CME-treated mice. This suggests that CME enhances the hepatic antioxidant defense system, thereby reducing oxidative stress and subsequent liver injury. The preservation of Nrf2/SOD activity underscores the potential of CME as an antioxidant and anti-inflammatory agent in the context of NAFLD/NASH.

Interestingly, the study also observed an increase in the liver-to-body mass (L/B) ratio in CME-treated mice. This change needs to be carefully interpreted. An increased L/B ratio could be indicative of hepatomegaly (liver enlargement), which is typically a negative outcome associated with liver pathology such as inflammation, fibrosis, or fatty liver disease. However, in the context of this study, the increase in L/B ratio may reflect positive changes in liver health. If the histopathological analysis shows reduced lipid accumulation and inflammation, the larger liver size could be due to healthier tissue, possibly resulting from increased protein content and improved metabolic activity. Therefore, the increased L/B ratio in CME-treated mice could represent a positive outcome of enhanced liver function and reduced pathological changes. The study’s results align with previous research indicating the beneficial effects of cucurbitacins and other phytochemicals in liver disease models. For example, cucurbitacin E has been shown to exert anti-inflammatory effects by inhibiting NF-κB signaling and reducing the production of pro-inflammatory mediators in various inflammatory conditions. Similarly, phenolic acids and flavonoids have been reported to exert hepatoprotective effects through their antioxidant and anti-inflammatory properties. The combined presence of these bioactive compounds in CME likely contributes to its overall therapeutic efficacy in NAFLD/NASH.

Despite these promising findings, there are limitations to this study that warrant further investigation. The precise molecular mechanisms underlying the observed effects of CME need to be elucidated through additional studies. For instance, detailed analyses of signaling pathways involved in lipid metabolism, inflammation, and oxidative stress are necessary to fully understand the therapeutic potential of CME. Future research should focus on optimizing the dosage regimens of CME and evaluating its long-term effects on liver health. Additionally, exploring the potential synergistic effects of CME with other therapeutic agents could provide new insights into combination therapies for NAFLD/NASH. Investigating the bioavailability and pharmacokinetics of cucurbitacin glycosides and other phytochemicals in CME will also be crucial for developing effective dietary supplements or pharmaceutical formulations.

## 5. Conclusions

In conclusion, our study provides insights into the mechanisms underlying the protective effects of CME against MCD-induced NAFLD. The observed downregulation of lipogenic genes, coupled with the upregulation of lipolytic genes, indicates that CME may modulate fatty acid metabolism, facilitating the clearance of lipids from the liver. Furthermore, the marked reduction in pro-inflammatory cytokines and preservation of *Nrf2/SOD* activity underscores the potential anti-inflammatory and antioxidant properties of CME. These findings serve as a foundation for future research aimed at unraveling the specific molecular targets and signaling pathways involved in the therapeutic actions of CME. Although our study established the promising therapeutic potential of CME in an MCD-induced NAFLD mice model, further investigation is warranted to comprehensively understand its mechanisms and facilitate the translation of these findings into clinical applications. Future studies should prioritize optimizing the dosage regimens, evaluating their long-term safety and efficacy in relevant animal models, and exploring their potential applications in human clinical trials.

## Data Availability

The original contributions presented in the study are included in the article/Supplementary Material, further inquiries can be directed to the corresponding author.

## Supplementary Materials

The following supporting information can be downloaded at: https://www.mdpi.com/article/10.3390/foods13132101/s1, Table S1. Primer sequences for RT-qPCR.
