# Supplementary material for: Citrullus mucosospermus Extract Exerts Protective Effects against Methionine- and Choline-Deficient Diet-Induced Nonalcoholic Steatohepatitis in Mice
Source: Foods. 2024 Jul 1;13(13):2101. doi: 10.3390/foods13132101 (PMC11240977; doi:10.3390/foods13132101)
Supplement: Supplementary file 1 [file foods-13-02101-s001.zip › foods-3068227-supplementary.pdf]

Supplement Table S1. Primer sequences for RT-qPCR

Primer name	Sequence (from 5' to 3')
TNFα	
Forward	CCT GTA GCC CAC GTC GTA
Reverse	TTG ACC TCA GCG CTG ACT TG
IL-6	
Forward	CTC TCT GCA AGA GAG TTC CAT CCA G
Reverse	GCT ATG GTA CTC CAG AAG ACC AGA GG
IL-1β	
Forward	AGG CTT CCT TGT GCA AGT GT
Reverse	TGA GTG ACA CTG CCT TCC TG
TGFβ	
Forward	GAG GTC ACC CGC GTG CTA
Reverse	TGT GTG AGA TGT CTT TGG TTT TCT C
C/EBPα	
Forward	GTG GAC AAG AAC AGC AAC GAG TAC
Reverse	GGA ATC TCC TAG TCC TGG CTT GC
PPARγ	
Forward	GAG TTC ATG CTT GTG AAG GAT GCA AGG
Reverse	CAT ACT CTG TGA TCT CTT GCA CG
FAS	
Forward	GAT CCT GGA ACG AGA ACA CGA TCT GG
Reverse	AGA CTG TGG AAC ACG GTG GTG GAA CC
aP2	
Forward	GAA CCT GGA AGC TTG TCT CCA GTG
Reverse	GAT GCT CTT CAC CTT CCT GTC GTC TGC
GAPDH	
Forward	GAG ACA GCC GCA TCT TCT TGT
Reverse	CAC ACC GAC CTT CAC CAT TTT
